# Periostin expression in uninvolved skin as a potential biomarker for rapid cutaneous progression in systemic sclerosis patients: a preliminary explorative study

**DOI:** 10.3389/fmed.2023.1214523

**Published:** 2024-01-24

**Authors:** Giacomo De Luca, Corrado Campochiaro, Samuele E. Burastero, Marco Matucci-Cerinic, Claudio Doglioni, Lorenzo Dagna

**Affiliations:** ^1^Unit of Immunology, Rheumatology, Allergy and Rare Diseases, IRCCS San Raffaele Hospital, Milan, Italy; ^2^School of Medicine, Vita-Salute San Raffaele University, Milan, Italy; ^3^Unit of Cellular and Molecular Allergology, IRCCS San Raffaele Scientific Institute, Milan, Italy; ^4^Division of Rheumatology AOUC, Department of Experimental and Clinical Medicine, University of Florence, Florence, Italy; ^5^Unit of Pathology, IRCCS San Raffaele Scientific Institute, Milan, Italy

**Keywords:** systemic sclerosis, scleroderma (or systemic sclerosis), periostin (POSN), skin fibrosis, fibrosis

## Abstract

**Objectives:**

This study aimed to evaluate periostin serum levels and skin expression in patients with systemic sclerosis (SSc).

**Methods:**

We enrolled 35 patients with diffuse (d-SSc) or limited (l-SSc) SSc, 15 patients with very early diagnosis of systemic sclerosis (VEDOSS), and 30 sex-matched healthy controls. Periostin serum levels were determined by an enzyme-linked immunosorbent assay (ELISA). Periostin skin expression was determined by immunohistochemistry (IHC) on paired involved and uninvolved 5-mm skin biopsy samples in a subgroup of 10 d-SSc and 10 L-SSc patients. A 12-month follow-up was considered.

**Results:**

We included 50 patients (mean age 53.1 ± 16.1 years; women 94%; mean disease duration 38.2 ± 45.1 months; anti-centromere 50%; anti-Scl70 40%), 35 of them with a definite SSc (68.8% l-SSc; 31.4% d-SSc; mean mRSS 9.0 ± 7.2) and 15 with VEDOSS; 30 controls were also included in this study. Periostin serum levels were higher in SSc patients compared to controls (32.7 ± 8.0 ng/mL vs. 27.7 ± 7.3 ng/mL; *p* < 0.001), while these levels were comparable among different groups of patients (29.7 ± 6.9 ng/mL in VEDOSS, 33.4 ± 7.8 ng/mL in lc-SSc; and 34.0 ± 8.5 in dc-SSc; *p* = ns). SSc patients with digital ulcers had higher periostin serum levels (36.2 ± 7.9 ng/mL vs. 30.6 ± 7.3 ng/mL, *p* < 0.02). Samples from the involved skin of l-SSc and d-SSc patients showed a significant dermal expression of periostin; an identical periostin expression was evident in the uninvolved skin of patients with d-SSc. In 7 out of 10 L-SSc patients, periostin expression was absent on uninvolved skin. In the remaining three l-SSc patients, a mild periostin expression on IHC was detectable on uninvolved skin and all of these three l-SSc patients presented a dramatic skin progression.

**Conclusion:**

Periostin skin expression may be a useful biomarker to indicate the presence of a disease at a higher risk of rapid cutaneous involvement.

## Introduction

Systemic sclerosis (SSc) is a connective tissue disorder with unknown etiology, characterized by microvascular abnormalities, immune dysregulation, and excessive deposition of collagen and other extracellular matrix (ECM) proteins, eventually resulting in fibrosis of the skin and internal organs (1). Among ECM mediators, cytokine transforming growth factor-beta 1 (TGF-β1) has been historically identified as a master regulator of the disease process in SSc since it accelerates fibrosis in the skin by inducing collagen production (2, 3). Despite much effort, the pathogenic mechanisms responsible for skin fibrosis remain elusive (3). Therefore, the management of skin fibrosis is still undefined, and no reliable biomarkers exist in a clinical practice to predict skin disease progression. Recently, a class of ECM proteins indicated as “matricellular proteins” has attracted increasing attention. These proteins specifically regulate cell–matrix interactions and play critical roles in tissue repair and fibrosis (4). Among them, periostin, a 90-kDa matricellular protein first identified 20 years ago as osteoblast-specific factor-2 (4), is mainly produced by fibroblasts (5–9). Periostin is expressed in many collagen-rich tissues and possesses important biological functions in the ECM, being prominently upregulated in adults during ECM remodeling and fibrosis (10–14).

Periostin, indeed, binds to collagen during fibrillogenesis, thus affecting the diameter of collagen fibers and the extent of cross-linking (4). Moreover, periostin binds to other ECM proteins, thereby organizing the ECM architecture, and serves as a ligand for integrins αvβ3 and αvβ5, which have been demonstrated to be upregulated in SSc fibroblasts (11, 12). Such signals can mediate cell adhesion to the ECM and may regulate certain cellular behaviors, including intracellular signaling, proliferation, and differentiation (14). The major producers of periostin are fibroblasts (5, 6), and its expression is induced by various factors, including TGF-β1, IL-4, and IL-13 (6, 7).

The potential involvement of periostin in the pathogenesis of skin fibrosis in SSc was initially suggested in a bleomycin (BLM)-induced skin fibrosis mouse model (15). Following BLM-induced skin fibrosis, the periostin null (PN−/−) mice showed significantly reduced dermal thickness and collagen deposition, indicating a reduction in the fibrotic process (15). Consistently, TGF-β-induced myofibroblast differentiation was attenuated in PN−/− fibroblasts *in vitro*, and although periostin by itself did not stimulate α-SMA expression in wild-type (WT) fibroblasts *in vitro*, TGF-β-induced α-SMA expression was enhanced in the presence of periostin. Thus, it is likely that periostin may act as a co-factor or as an enhancer of TGF-β bioactivity, creating a favorable environment for the action of fibrogenic-3 factors. Preliminary studies reported the enhanced expression of periostin in the clinically involved skin of SSc patients with diffuse disease (15, 16). Along this line, recently, a significant elevation of serum periostin levels in 56 patients with SSc was reported (16) and correlated with the severity of skin fibrosis.

This study aimed to evaluate periostin serum levels and periostin skin expression as potential prognostic biomarkers in SSc patients.

## Methods

This project was a monocentric prospective study on a cohort of SSc patients followed up at our tertiary scleroderma unit at IRCCS San Raffaele Hospital in Milan (Italy). Enrolled patients and control subjects signed a written informed consent. The study protocol was in accordance with the Declaration of Helsinki and approved by our institutional review board.

### Patients and methods

Patients fulfilling the 2013 ACR/EULAR classification criteria for SSc (17), patients classified as very early diagnosis of systemic sclerosis (VEDOSS) (18), and sex- and age-matched healthy controls were enrolled. A comprehensive evaluation of disease characteristics and organ involvement was performed in SSc and VEDOSS patients, including disease subtype, disease duration, autoantibody profile, skin involvement as evaluated by a modified Rodnan skin score (mRSS), and assessment of digital ulcers and disease-related internal organ involvement (i.e., chiefly, lung, heart, kidney, and gastrointestinal organs). Pulmonary function tests (PFTs) with the evaluation of % predicted forced vital capacity (% pFVC) and % predicted diffusing capacity for carbon monoxide (% pDLCO), high-resolution computed tomography (HRCT), and echocardiography were performed at baseline in all patients. Additional tests, such as 24 h-ECG Holter, cardiac magnetic resonance, or other instrumental examinations, were performed on a case-by-case basis when clinically recommended. Data on concomitant therapies and comorbidities were also recorded for the entire cohort.

The SSc patients were classified according to the extent of skin thickness (limited cutaneous disease [lc-SSc] vs. diffuse cutaneous disease [dc-SSc]) as well as according to the presence of major organ involvement. The appearance of the first non-Raynaud’s manifestation was considered disease onset and was used to calculate disease duration.

### Periostin serum levels

At baseline, periostin serum levels were measured in the enrolled patients and in 30 sex- and age-matched healthy controls. Blood was taken in the morning after 12 h of fasting, and sera was immediately stored at −80°C at our institutional biobank until assayed. Periostin serum levels were measured by a commercial sandwich enzyme-linked immunosorbent assay (ELISA) (an analytical sensitivity of 80 pg./mL) according to the manufacturer’s protocol (Thermo Fisher Scientific, Waltham, MA, USA).

### Periostin skin expression

In a subgroup of 20 SSc patients, periostin skin expression was evaluated by immunohistochemistry (IHC) on 5 mm skin punch-biopsy samples, specifically in involved (*forearm*) and uninvolved (*gluteus*) skin samples in 10 patients with dc-SSc and in 10 patients with lc-SSc. All patients gave written informed consent to the procedure. Formalin-fixed, paraffin-embedded blocks were sectioned at 5-μm thickness and immunostained for periostin with the Ventana Benchmark automated staining system (Ventana-Roche Medical Systems, Tuscon, AZ, USA). Briefly, slides were deparaffinized, rehydrated, quenched for endogenous peroxidase, subjected to heat-induced antigen retrieval with pH6 citrate buffer, CC1, and incubated with a rabbit polyclonal antibody (dil.1:500, cod. RD181045050, BioVendor Lab, Modrice, Cz). The immune reaction was detected with the UltraVIEW™ DAB detection kit, and the slides were counterstained with hematoxylin.

With the same automated staining system, the same skin samples were immunostained for α-SMA (myofibroblasts), PECAM-1 (endothelial cells), CD3+ (leukocytes), CD68+ (macrophages), CD14+ (monocytes), and CD163+ (M2-polarized macrophages). To quantify collagen accumulation in the dermis, we employed the Image J software (freely downloadable and developed by Wayne Rasband at the Research Services Branch of the NIH) according to a previously described method (19). Collagen deposition was separately examined for the papillary and reticular dermis and was correlated with mRSS and both tissue periostin and serum periostin levels to evaluate whether these latest findings may have any correlation with the degree of skin fibrosis.

### Follow-up

Data obtained at the time of blood tests for periostin and, for a subgroup of 20 patients, at the time of skin biopsy were used for the determination of the length of a follow-up of at least 12 months.

During the follow-up, each patient underwent a complete clinical assessment every 3 months with mRSS: The skin progression was defined as an increase of >10% in mRSS despite appropriate therapy (methotrexate, mycophenolate mofetil, or cyclophosphamide, also combined with rituximab in selected cases) or worsening of mRSS of >30% in untreated patients, or an increase of >5 points regardless of therapy, as previously described (20). Additional laboratory or instrumental evaluations were prescribed when clinically indicated.

### Statistical analysis

Data were analyzed using SPSS 24.0 (SPSS, Chicago, IL). Continuous variables were reported as mean ± standard deviation, while categorical variables were reported as number and percentage. The Mann–Whitney U-test or Wilcoxon’s rank sum test, as appropriate, was used to compare continuous variables; a value of *p* of <0.05 was considered statistically significant.

To evaluate the skin score progression in all patients, categorical variables of change (worse, stable, and improved) were made by combining significant changes in mRSS. Changes in mRSS during the follow-up were correlated with baseline periostin serum levels.

## Results

Our cohort consisted of 35 SSc patients according to 2013 ACR/EULAR classification criteria and 15 patients diagnosed with VEDOSS; 30 sex- and age-matched healthy controls were also included in this study.

Demographic and clinical characteristics of enrolled patients are summarized in Table 1.

**Table 1:** Demographic and clinical characteristics of patients.

Characteristic	All patients (50)*	SSc Patients (35)**
Age (years, mean ± SD)	53.25 ± 17.1	53.1 ± 16.1
Female patients, *n* (%)	48 (96)	33 (94)
Disease duration (months, mean ± SD)	N/A	38.2 ± 45.1
Limited cutaneous SSc, *n* (%)	N/A	24 (68.6)
Modified Rodnan skin score (mean ± SD)	N/A	9.0 ± 7.2
Anti-centromere antibodies positivity, *n* (%)	25 (50)	13 (37.1)
Anti-Scl70 antibodies positivity, *n* (%)	20 (40)	19 (54.3)
ANA positivity without SSc Ab, *n* (%)	2 (4)	0 (0)
Other SSc-related antibodies positivity	3 (6)	3 (8.6)
Patients with a history of digital ulcers, *n* (%)	N/A	16 (45.7)
Patients with active or recurrent digital ulcers, *n* (%)	N/A	4 (11.4)
History of calcinosis, *n* (%)	N/A	6 (17.1)
Gastrointestinal involvement, *n* (%)	N/A	11 (31.4)
Musculoskeletal involvement, *n* (%)	N/A	5 (14.3)
ILD on HRCT, *n* (%)	N/A	14 (40)
Decrease in DLCO on PFTs, *n* (%)	N/A	22 (62.9)
DLCO, % (mean ± SD)	N/A	76.0 ± 17.1
Decrease in FVC on PFTs, *n* (%)	N/A	4 (11.4)
FVC, % (mean ± SD)	N/A	103.9 ± 18.2
Pulmonary arterial hypertension, *n* (%)	N/A	0 (0)
PASP on echocardiography, mmHg (mean ± SD)	N/A	26.2 ± 5.6
Myocardial involvement, *n* (%)	N/A	5 (14.3)
Elevation of cardiac biomarkers, *n* (%)	N/A	13 (37.1)
Scleroderma pattern at NVC, *n* (%)	50 (100)	35 (100)
Calcium-antagonist therapy, *n* (%)	50 (100)	35 (100)
Intravenous iloprost therapy, *n* (%)	0 (0)	7 (20)
PDE5-inhibitor therapy, *n* (%)	0 (0)	5 (14.3)
Bosentan therapy, *n* (%)	0 (0)	2 (5.7)
Methotrexate therapy, *n* (%)	N/A	17 (48.6)
Mycophenolate mofetil therapy, *n* (%)	N/A	12 (34.3)

*Including patients fulfilling the 2013 ACR/EULAR classification criteria and patients classified as VEDOSS. **Only patients fulfilling the 2013 ACR/EULAR classification criteria. N, number; SD, standard deviation; Ab, antibodies; ILD, interstitial lung disease; HRTC, high-resolution computed tomography; DLCO, diffusing capacity for carbon monoxide; PFTs, pulmonary function tests; FVC, forced vital capacity; NVF, nailfold videocapillaroscopy; PDE5, phosphodiesterase-5.

The mean age of our patients was 53.1 ± 16.1 years, and they were almost all women (*n* = 33, 94%), with a mean disease duration of 38.2 ± 45.1 months. In total, 35 patients had a definite SSc; among them, 24 patients presented a limited cutaneous involvement (68.6%), while 11 patients (31.4%) had a diffuse involvement. The mean mRSS at baseline was 9.0 ± 7.2.

Considering the entire cohort of 50 SSc patients, anti-centromere (ACA) was found in 25 cases (50.0%), while 20 patients were anti-Scl70 positive (40%); 5 patients (6.0%) were positive for other SSc-related antibodies (i.e., anti-RNA-III and anti-PM/Scl); and 2 patients (4.0%) showed only ANA positivity.

### Periostin serum levels in patients and controls at baseline

Periostin serum levels were higher in SSc patients compared to controls (32.7 ± 8.0 ng/mL vs. 27.7 ± 7.3 ng/mL, *p* < 0.001), while they were comparable in different groups of patients (29.7 ± 6.9 ng/mL in patients with VEDOSS; 33.4 ± 7.8 ng/mL in patients with lc-SSc; 34.0 ± 8.5 in patients with dc-SSc; *p* = ns for all comparisons). Specifically, no differences in periostin serum levels emerged with respect to disease subtype, disease duration, presence and extent of organ involvement, autoantibody profile, and current or previous treatment. Periostin serum levels did not correlate with the extent of skin involvement measured by mRSS (*data not shown*). However, higher periostin serum levels were found in SSc patients with an active nailfold videocapillaroscopy and a history of digital ulcers (36.2 ± 7.9 ng/mL) when compared to other patterns without digital ulcers (30.6 ± 7.3 ng/mL) (*p* < 0.02).

### Periostin skin expression on IHC: baseline data

Periostin skin expression was evaluated in 20 SSc patients (10 lc-SSc and 10 dc-SSc; female patients 17 [85%]; mean age 52.1 ± 12.8 years) who gave written informed consent to the procedure of punch skin biopsy.

**Figure 1:**
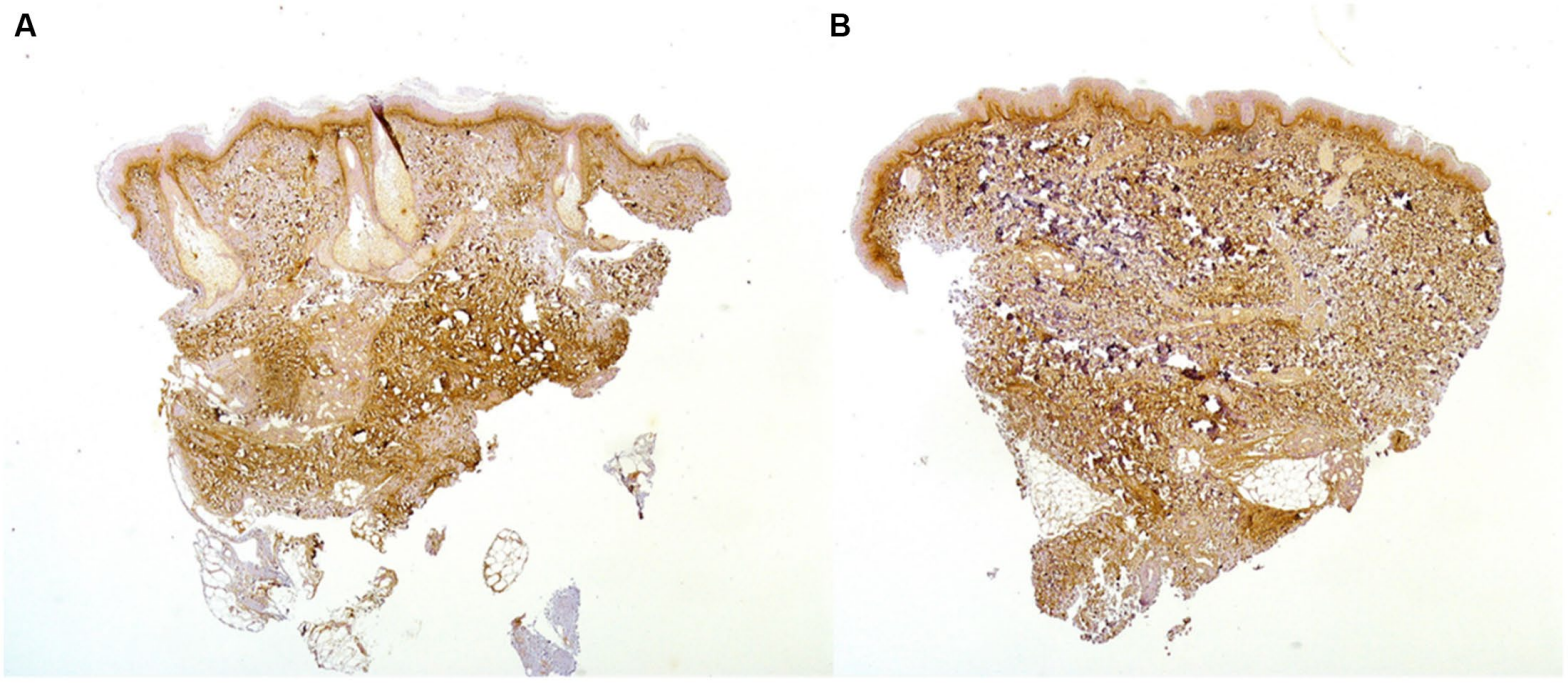
Skin biopsy of a 43-year-old patient with anti-Scl70+ dc-SSc shows a high expression of periostin in the upper dermis and a robust periostin staining in the fibrotic and inflammatory areas of the lower dermis in both involved **(A)** and clinically uninvolved **(B)** skin (20x).

On IHC, skin samples from involved SSc patients with both lc-SSc and dc-SSc showed a remarkably high expression of periostin in the upper dermis and robust periostin staining in the fibrotic and inflammatory areas of the lower dermis, mainly infiltrated by lymphocytic and macrophages. Interestingly, a similar immunohistochemical expression of periostin was evident in the uninvolved skin of patients with dc-SSc (*representative image* in Figure 1).

**Figure 2:**
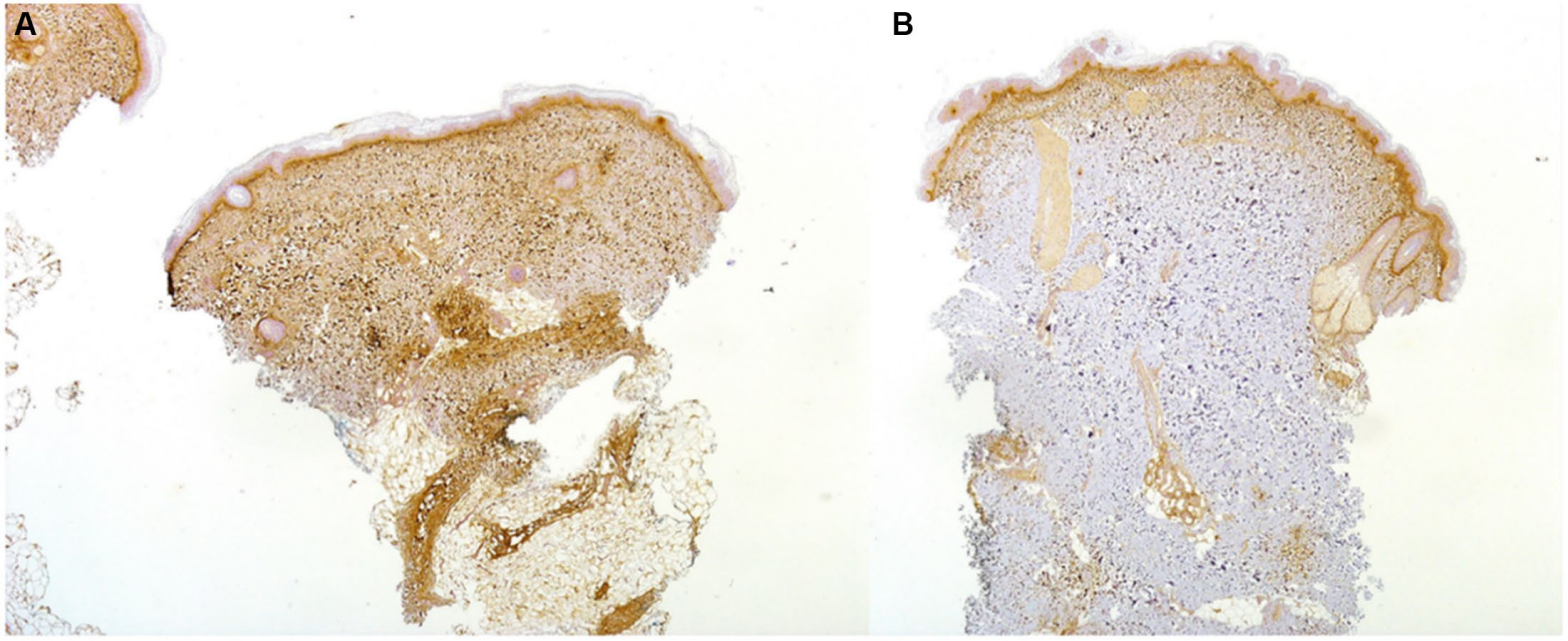
Skin biopsy of a 37-year-old patient with ACA+ lc-SSc demonstrating a high expression on IHC (20x) of periostin in the upper dermis and in the fibrotic and inflammatory areas of the lower dermis of the involved skin **(A)**: periostin was absent on IHC in the uninvolved skin **(B)**.

Conversely, in 7/10 lc-SSc patients, periostin expression was completely absent in uninvolved skin (*representative image* in Figure 2), and the absence of periostin appeared as the only notable difference on IHC between involved and uninvolved skin samples in those lc-SSc patients. A similar distribution of a-SMA+ cells, endothelial cells, and CD3+ and CD68+ cells was detected in both affected and unaffected skin samples (*data not shown*).

**Figure 3:**
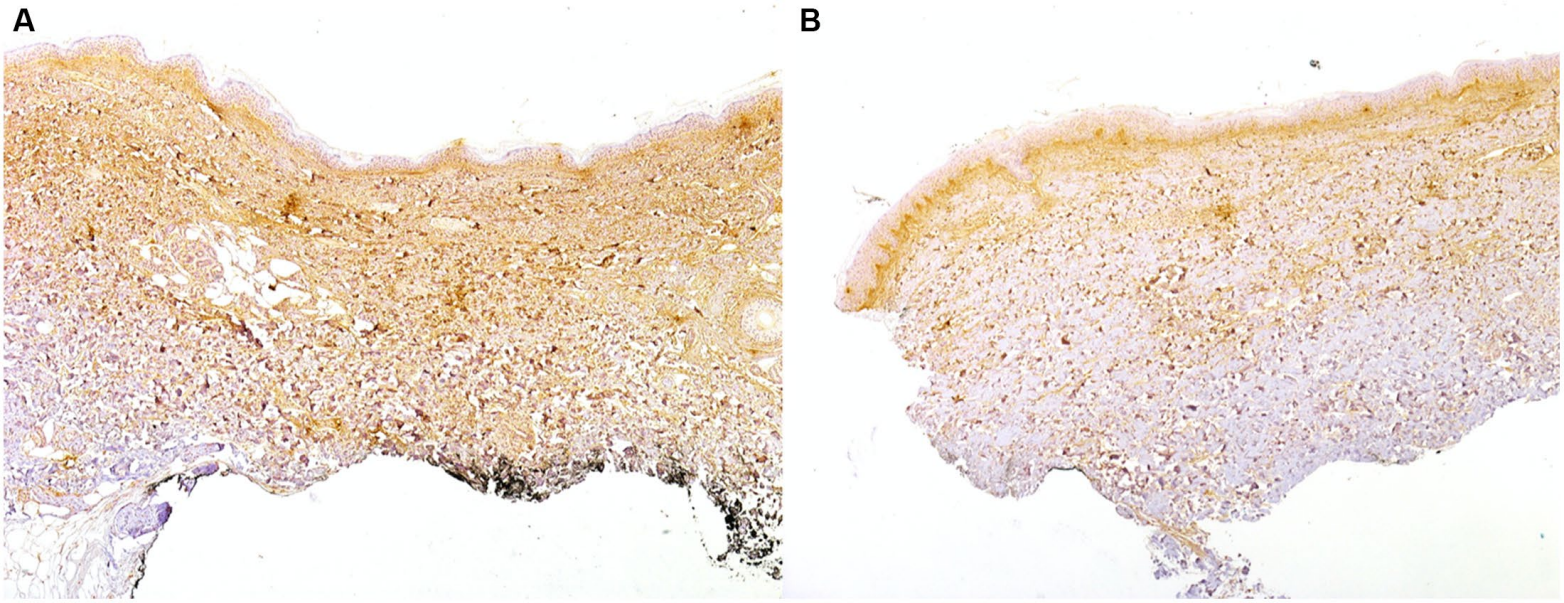
IHC on skin biopsy of a 63-year-old patient with ACA+ early (< 3 years disease duration) and progressive lc-SSc showing a high expression of periostin in the upper dermis and in the fibrotic and inflammatory areas of the lower dermis involved **(A)** and uninvolved **(B)** skin (20x).

In the remaining three lc-SSc ACA+ patients, a mild periostin dermal expression on IHC was detectable. Periostin skin expression was the only IHC differential finding in the uninvolved skin between dc-SSc and lc-SSc patients (*representative image* in Figure 3).

### Follow-up data

Considering the entire cohort, none of the 15 patients with VEDOSS progressed during the 12-month follow-up. Among the 35 SSc patients, during the 12-month follow-up, 4 of them (11.4%) showed a skin disease progression, as previously defined, presenting an increase of >30% in mRSS.

No significant differences in periostin serum levels at baseline were noted between patients with different cutaneous disease progression (31.4 ± 6.3 ng/mL in patients who improved, 32.3 ± 7.1 ng/mL in patients who maintained a stable mRSS; and 33.1 ± 7.8 in patients whose mRSS worsened during the follow-up; a value of *p* of >0.05 for all comparisons).

**Figure 4:**
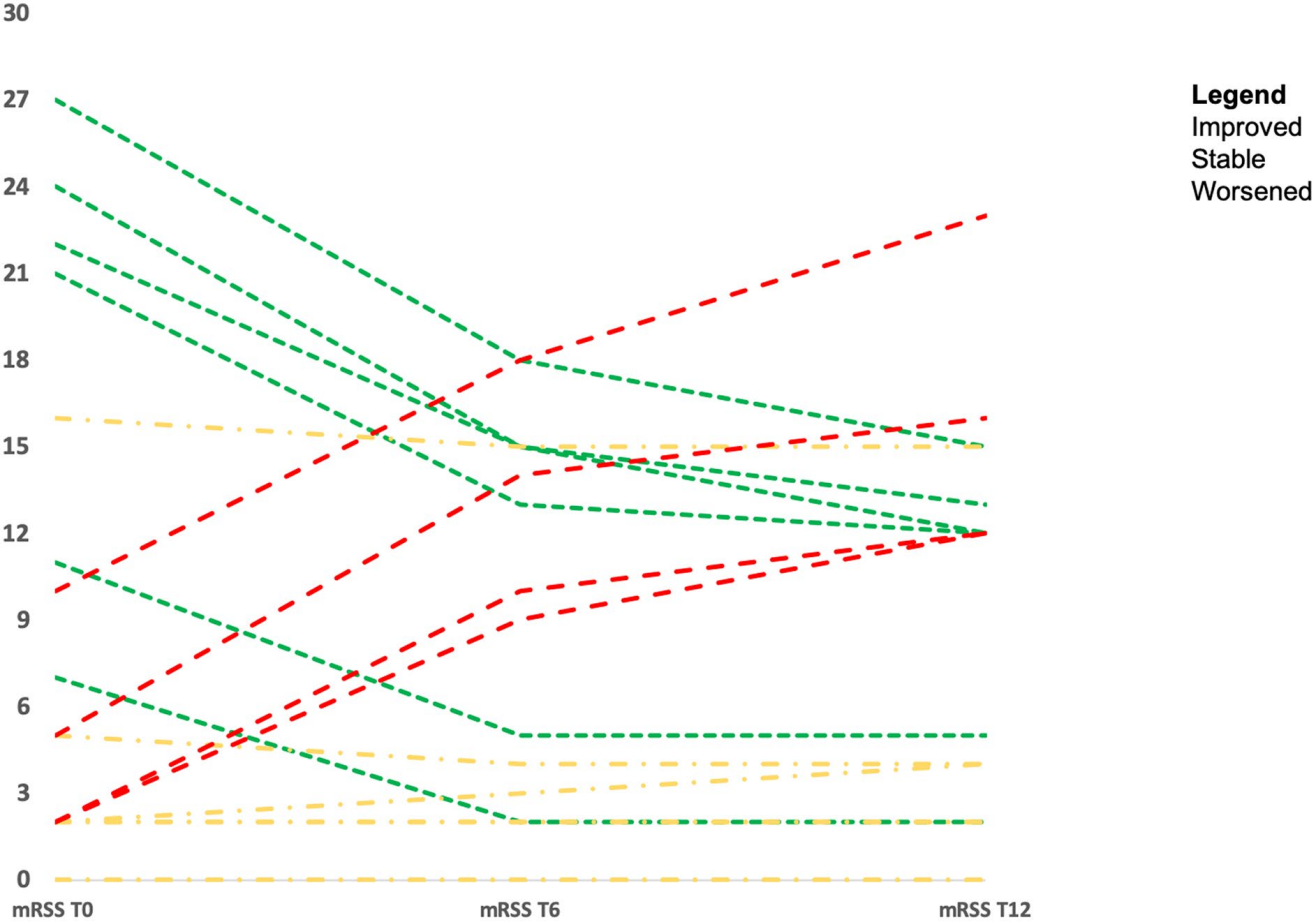
Modified Rodnan skin score over time in 20 SSc patients who underwent skin biopsy.

Considering the 20 SSc patients who underwent skin biopsy, the majority of them improved with therapy or remained stable during the follow-up. Eight patients (40.0%) showed a significant decrease in mRSS of a minimum of 5 points (mRSS from 18.6 ± 7.5 at baseline vs. 9.8 ± 7.1 at 12 months, *p* < 0.001) and in eight patients (40.0%) mRSS values remained stable (mRSS from 3.2 ± 5.3 at baseline vs. 3.4 ± 4.5 at 12 months; *p* = ns). The remaining four patients (one with dc-SSc treated with MMF and three untreated patients with lc-SSc) (20.0%) showed a significant worsening of skin thickness (mRSS from 4.8 ± 3.5 at baseline vs. 15.8 ± 4.5 at 12 months; *p* < 0.001). Changes in mRSS over time in the 20 SSc patients who underwent skin biopsy are represented in Figure 4.

Interestingly, all three lc-SSc patients (ACA+ and untreated with immunosuppressants, due to limited diseases) with mild periostin IHC expression on uninvolved skin presented a skin progression with >30% increase in the mRSS. However, in the seven patients affected by lc-SSc and without periostin skin expression, a stability of the mRSS during the follow-up was observed, even though the difference was not statistically significant (*p* = 0.74), due to the low number of patients and events.

## Discussion

In our observational study, we found increased periostin serum levels in SSc patients and differential periostin skin expression in uninvolved skin among patients with diffuse and limited cutaneous disease. Our preliminary results suggest that periostin dermal expression on uninvolved skin might, in the clinical practice, herald a higher risk of a progressive disease characterized by rapid cutaneous progression. Once confirmed in prospective studies and in larger cohorts, assessment of periostin skin expression could be proposed as a potential useful tool to identify patients at a higher risk of rapid cutaneous progression, thus making them eligible for induction therapy to obtain an early disease remission.

It is well known that SSc is a rare life-threatening autoimmune disease with a poor prognosis and a high mortality risk (21). The majority of deaths occur due to disease progression, in particular for progressive lung or heart SSc-related involvement (21). In the clinical practice, the identification of patients at a higher risk of life-threatening events and with poor prognosis is the major goal. Data from the EULAR Scleroderma Trials and Research (EUSTAR) database indicate that more extensive skin and lung involvement at baseline are both associated with decreased survival in SSc patients (21). In diffuse SSc, the skin thickness progression rate predicts mortality and early internal organ involvement (22). In this scenario, the identification of prognostic biomarkers able to predict the skin progression rate is eagerly needed. Periostin, belonging to the class of “matricellular proteins,” has recently attracted increasing attention in the field of various fibrotic processes (2–4, 13), including SSc (3).

In SSc, elevated IL-13 circulating levels, the main inducer of periostin, have been found (8, 9), supporting the existence of a biological loop. Indeed, evidence supports the pivotal role of type 2 inflammation in SSc pathogenesis, with increased T-helper type 2 (Th2)-polarized cell tissue infiltration and higher levels of circulating IL-4 and IL-13 (23). Of relevance, periostin is an IL-4/IL-13-inducible gene and a potential surrogate marker of Th2 inflammation (7). Periostin was, thus, recently used as a biomarker in a proof-of-concept study in SSc patients to assess the response to romilkimab (a monoclonal bispecific antibody against IL-4/IL-13), showing a trend toward reduction compared to placebo (24).

Previously, two preliminary studies reported an enhanced expression of periostin in the skin of 18 SSc patients (15, 16): a more intense deposition of periostin was noted in the clinically affected dc-SSc skin compared to lc-SSc patients. Periostin was also colocalized within fibroblasts, α-SMA+ myofibroblasts, and endothelial cells. The involvement of periostin in the pathogenesis of skin fibrosis in SSc was then confirmed using a BLM-induced skin fibrosis mouse model (16).

To better assess the role of periostin in fibrosis, a Japanese group evaluated levels of periostin in skin and lung SSc fibroblasts (23). Levels of ECM proteins and pro-fibrotic factors were evaluated in periostin-expressing human skin fibroblasts in the presence or absence of TGF-β. The effects of periostin on the SMAD proteins were also evaluated following stimulation with TGF-β by immunoblotting, immunofluorescence staining, and RNA interference. Periostin was strongly expressed in skin and lung fibroblasts from SSc patients. Although recombinant periostin alone did not affect ECM protein levels, TGF-β and recombinant periostin treatment or periostin overexpression in skin fibroblasts significantly enhanced the production of ECM proteins. Overexpression of periostin in the presence of TGF-β also augmented expressions of α-SMA and early growth response-1 but decreased the level and activity of matrix metalloproteinase-1. Interestingly, the level of SMAD-7, a TGF-β-inducible inhibitor of TGF-β signaling, was reduced in periostin-expressing fibroblasts but increased in periostin-silenced fibroblasts. In addition, SMAD-7 reduction induced by periostin was partially inhibited in integrin αV-silenced fibroblasts. Thus, these results suggest that periostin contributes to fibrosis by enhancing TGF-β signaling via SMAD-7 inhibition, which may lead to ECM deposition and periostin generation (25).

Recently, serum periostin levels had been found to be considerably higher in the early stage of dc-SSc and were intensely correlated with the severity of skin fibrosis as determined by the mRSS (15). Surprisingly, despite evidence that periostin might be a useful marker for idiopathic pulmonary fibrosis (26), no association with SSc-ILD was found (15).

In another recent study, periostin levels were increased in SSc patients and directly correlated with mRSS and echocardiography parameters of left ventricular measurements (27). The levels of periostin were assessed in the serum of 106 SSc patients and 22 healthy controls and assessed by immunofluorescence staining in cardiac tissue from 4 SSc patients and 4 controls. Immunofluorescence staining in SSc cardiac tissue showed patchy periostin expression but not in controls; there was also extensive periostin expression in areas without collagen deposition, while all established fibrotic areas showed colocalization of collagen and periostin. No association between periostin levels and interstitial lung disease, pulmonary hypertension, or other vascular complications was detected (27).

In our cohort, the increased periostin serum levels did not differ among SSc subsets based on disease duration, presence and extent of skin involvement, autoantibody profile, or current or previous treatment. The reason for this lack of correlation between periostin serum levels and mRSS in our cohort may depend on several factors. We speculate that a role could be played by the small sample size and by the relatively low degree of skin fibrosis in our cohort (mean mRSS <10), mainly composed of patients with an early disease. Indeed, 15 patients with VEDOSS and without skin fibrosis were also enrolled in our study. Conversely, the study by Yang et al. (15) and the study by El-Adili et al. (27) included patients with early d-SSc and definite SSc with a long disease duration (12 years), respectively. Interestingly, higher periostin serum levels were found in SSc patients with an active pattern on nailfold videocapillaroscopy and a history of digital ulcers. These results are in line with the role of periostin in wound healing and epithelial-mesenchymal transition and deserve future discussion.

In our study, in dc-SSc, a remarkably high expression of periostin on IHC was detected in the upper dermis as well as in the fibrotic and inflammatory areas of the lower dermis, in both involved and uninvolved skin samples. Periostin expression was completely absent on uninvolved skin in 7 out of 10 lc-SSc patients. The lack of periostin expression was the only visible difference in IHC between involved and uninvolved skin samples in patients affected by lc-SSc. The uninvolved skin of three lc-SSc patients, however, showed a mild periostin expression on IHC, and all three lc-SSc patients (ACA+ and untreated due to a limited cutaneous and not complicated disease) with mild periostin IHC expression on uninvolved skin presented a significant skin progression with >30% increase in the mRSS during the follow-up. Conversely, the seven lc-SSc patients without periostin skin expression presented a stable mRSS after 6 months.

Based on these considerations, we may suggest that periostin skin expression evaluated by IHC on clinically unaffected skin may differentiate patients with dc-SSc from those with lc-SSc. Moreover, it may, among patients initially classified as affected by a limited cutaneous disease, be those at a higher risk of cutaneous progression and, thus, at a higher risk of disease progression.

Thus, once confirmed in prospective studies on a larger cohort of SSc patients, periostin expression on uninvolved skin could be proposed as a “prognostic biomarker” able to predict skin progression rate and guide a prompt therapeutic intervention.

The upcoming availability of therapeutic strategies directed against periostin could pave the way to a pathogenic-based targeted therapy. Interestingly, direct administration by the intranasal route of small interfering RNA (siRNA) or antisense oligonucleotide against periostin into the lungs of a BLM-induced experimental model of pulmonary fibrosis was associated with significantly reduced levels of periostin and TGF-β1 in airway fluid and lung tissue, as well as a reduced deposition of collagen in lung tissue and a decrease in the lung fibrosis score in treated mice compared to control mice (28). These results are consistent with the demonstration of increased serum levels of periostin in patients with idiopathic interstitial pneumonias, which reflected histopathological classifications and pulmonary function (29).

To the best of our knowledge, this is the first study evaluating the possible role of periostin skin expression as a prognostic biomarker in the clinically uninvolved skin of SSc patients.

Our study has several limitations. First, the small sample size, with particular reference to the number of patients with diffuse SSc and to the low number of patients who presented a skin disease progression, represents a major limitation of the study and limits the possibility for any generalizability of the findings. Considering such a small sample size, our preliminary study needs to be solely considered the potential first piece for future larger prospective studies. Second, the short follow-up did not allow to identify patients with slower disease progression, preventing any definite conclusion on the role of periostin as a prognostic biomarker. Third, the evaluation of periostin skin expression on skin samples by IHC was based on a semi-quantitative assessment since no specific recommendations or thresholds are available, and *in situ* hybridization was not performed, thus potentially introducing subjectivity in the results. Finally, no data on lung or heart disease progression were available.

In conclusion, our data need to be verified in larger SSc cohorts with a longer follow-up evaluating periostin circulating levels and its expression on involved and uninvolved skin samples to identify those patients that are at a higher risk of cutaneous progression.

## Data availability statement

The raw data supporting the conclusions of this article will be made available by the authors, without undue reservation.

## Ethics statement

The studies involving humans were approved by IRCCS Ospedale San Raffaele, Milan (Italy). The studies were conducted in accordance with the local legislation and institutional requirements. The participants provided their written informed consent to participate in this study.

## Author contributions

GDL conceived the hypothesis, designed the experiments, and responsible for the clinical management of patients, performed the statistical analysis, and wrote the manuscript. CC responsible for clinical management of patients, statistical analysis, and revising the manuscript. SB responsible for assessment of periostin serum levels, statistical analysis, and revising and editing the manuscript. MM-C and LD revised and approved the final manuscript. CD did the experiments on skin biopsy (IHC), revised, and approved the final manuscript. All authors contributed to the article and approved the submitted version.
